# Changes in sST2 and NT-proBNP levels predict early cardiac arrhythmia in breast cancer patients treated with anthracycline-containing chemotherapies

**DOI:** 10.3389/fcvm.2024.1477679

**Published:** 2024-12-12

**Authors:** Cuncun Chen, Hui Zheng, Yanchun Wang, Ying Tong, Heng Zhang, Suhong Xie, Xiaolu Ma, Minglei Jiang, Zhiyun Gong, Tianqing Yan, Yanan Tian, Lin Guo, Renquan Lu

**Affiliations:** Department of Clinical Laboratory, Fudan University Shanghai Cancer Center, Shanghai, China

**Keywords:** soluble ST2, NT-ProBNP, cardiac toxicity, arrhythmia, chemotherapy

## Abstract

**Background:**

Cardiovascular biomarkers are crucial for monitoring cancer therapy-related cardiac toxicity, but the effects on early stage are still inadequate. To screen biomarkers in patients with breast cancer who receive anthracycline-containing chemotherapy, we studied the behavior of six biomarkers during chemotherapy and their association with chemotherapy-related cardiac toxicity.

**Methods:**

In a prospective cohort of 73 patients treated with anthracycline-containing chemotherapy, soluble suppression of tumorigenicity 2 (sST2), high-sensitivity cardiac troponin T, N-terminal pro-B-type natriuretic peptide (NT-proBNP), myoglobin, creatine kinase isoenzyme MB, and heart-fatty acid binding protein were measured at baseline, during chemotherapy cycle (C1–C6). According to whether arrhythmia occurred, patients were divided into two groups (healthy group or arrhythmias group), and basic clinical characteristics were collected and compared. Logistic regression analyses and receiver operating characteristic (ROC) curves were conducted to investigate the association between the changes in biomarkers and arrhythmia.

**Results:**

sST2 levels increased significantly from baseline to C1 (*P* < 0.01). NT-proBNP levels decreased from baseline to C1 and C5 (*P* < 0.01). The logistic regression analysis showed a greater risk of arrhythmia was associated with interval changes in sST2 [odds ratio (OR): 1.27; 95% CI: 1.03–1.56; *P* = 0.024] and NT-proBNP (OR: 0.83; 95% CI: 0.70–0.98; *P* = 0.029). The ROC curves showed that ΔsST2, ΔNT-proBNP, and ΔsST2 + ΔNT-proBNP had good predictive value for arrhythmia (areas under the curves were 0.631, 0.633, and 0.735, respectively, *P* < 0.05).

**Conclusions:**

Early changes in sST2 and NT-proBNP levels offer additive information for early arrhythmia prediction in breast cancer patients who receive anthracycline-containing chemotherapy.

## 1 Introduction

In 2020, breast cancer accounted for 11.7% of all new cancer cases globally, and the mortality rate reached 6.9% (1). Chemotherapy, radiotherapy, and endocrine therapy are commonly used as adjuvant treatments for breast cancer, and the mortality or recurrence is reduced by 10%–25% for most options. However, diseases caused by side effects of treatment seriously influence patients’ life quality and increase non-breast-cancer mortality (2). Commonly used cardiotoxic cancer therapies have resulted in a high prevalence of all-cause mortality and cancer therapy-related cardiovascular toxicity, especially in elderly patients and patients who receive anthracycline therapy (3). Cardio-oncology is an emerging research field in cancer therapy, with many other cardiovascular diseases, especially arrhythmias, accounting for an increasing proportion (4) in addition to cancer therapy-related cardiac dysfunction (CTRCD). Age, cancer history, previous risk of cardiovascular toxicity or diseases, and different treatment regimens including dose, frequency, and duration of chemotherapy are all factors influencing cardiac function (5). Thus, early identification, evaluation, and management of cardiovascular toxicity in cancer patients undergoing treatment are equally important to the treatment itself.

Chemotherapy drugs such as anthracyclines induce dose-dependent cardiac toxicity, leading to irreversible myocardial damage. Studies have shown that anthracyclines may induce myocardial oxidative stress and mitochondrial dysfunction, and lead to myocardial injury (6). Anthracyclines (such as epirubicin and doxorubicin) are also risk factors for arrhythmia (7). The incidence of CTRCD due to taxane therapy was reported to be only 0.7%, but the incidence increases up to 20% when combined with high-dose anthracyclines (8). The first case of bradycardia during cancer treatment was related to paclitaxel, with nearly 30% of patients experiencing bradycardia episodes (9).

In the current guidelines, echocardiographic assessment of left ventricular ejection fraction (LVEF) is recommended as a monitoring strategy (10). However, LVEF assessment is limited in the sensitive assessment of early, subclinical damage to cardiac function. Cardiac biomarkers are promising tools for the early detection and identification of cancer treatment-related cardiac toxicity (11). Troponins and natriuretic peptides [B-type natriuretic peptide (BNP)/N-terminal pro-B-type natriuretic peptide (NT-proBNP)] are the primary recommended blood biomarkers as diagnostic indicators for cardiac toxicity, and dynamic monitoring changes can predict cardiovascular toxicity after chemotherapy (5, 12, 13). A meta-analysis (14) of 61 trials with 5,691 patients, showed the high exclusive diagnostic value of troponin, and the negative predictive value for CTRCD was 93%. A prospective study (15) showed that high-sensitivity cardiac troponin T (hs-cTnT) and NT-proBNP levels increased in breast cancer patients treated with anthracycline drugs and/or trastuzumab drugs and were associated with an increased risk of CTRCD. However, the current research on troponins and BNP/NT-proBNP mainly focuses on the decrease in LVEF, with arrhythmia rarely studied.

The suppression of tumorigenicity 2 (ST2) protein is part of the interleukin 1 receptor family with the transmembrane (ST2l) and soluble (sST2) subtypes and binds to interleukin 33 (IL-33). sST2 could be released by cardiomyocytes in response to myocyte stretch or inflammation (16), and thus could be used as a valuable biomarker in heart failure. Few studies have predicted the value of sST2 for cancer treatment-related cardiac toxicity. In breast cancer patients using anthracycline drugs, sST2 levels significantly increased in cycle 2 of the treatment, similar to the changes in cTnT and NT-proBNP (17). In another study involving 126 breast cancer patients who received doxorubicin, trastuzumab, or combination therapy with a 6-month follow-up, the results showed that sST2 levels have a positive correlation with left ventricular volume and E/e’ ratio, but a negative correlation with LVEF (18). sST2 level may be related to cardiac toxicity induced by radiotherapy, and a 3-year follow-up showed increased sST2 levels were associated with global longitudinal strain (GLS) changes (19). Previous studies mainly focused on changes in biomarkers in cycles 2–4 at 3–6 months or longer after treatment. Few research studies have investigated the initial changes in biomarkers during cancer treatment.

To better identify the potential predictive value of these biomarkers for chemotherapy-related arrhythmia in breast cancer patients throughout the entire anthracycline-based treatment, we monitored the dynamic changes in multiple cardiovascular biomarkers in patients before and during an anthracycline-based treatment regimen and evaluated the associations of the changes in biomarkers with the risk of arrhythmia. However, according to real clinical investigations conducted by doctors, echocardiography of the LVEF is not regularly conducted during an anthracycline-based regimen due to its cost and complexity unless a patient presents with typical cardiovascular symptoms. An electrocardiogram (ECG) is performed before each chemotherapy process due to its convenience. Thus, as this research aimed to replicate the real clinical situation, we used electrocardiogram results to evaluate cardiac function.

## 2 Methods

### 2.1 Study population

The recruitment of study patients occurred prospectively at Fudan University Shanghai Cancer Center from August 2022 to September 2023. Patients aged 18 years and older with treatment-naive primary breast cancer were enrolled (*n* = 73, median age = 46.4 years, range = 40–54 years). The exclusion criteria included patients (1) who had undergone cardiac or vascular surgery or who had myocardial or vascular diseases within 3 months prior to enrollment, (2) who were pregnant, (3) who were treated for cancer recurrence, (4) who had breast cancer combined with other malignant tumors, and (5) who had serious infection. Patient and tumor characteristics are summarized in Table 1.

**Table 1:** Basic clinical characteristics of the overall cohort and stratified according to cardiac toxicity.

	All (*n* = 73)	Normal (*n* = 34)	Arrhythmia (*n* = 39)	*P*-value
Age (years)	46.4 (40–54)	48.7 (43–55)	44.3 (35.5–53.5)	0.112
Weight (kg0	57.4 (51.8–62)	56.2 (51–60)	58.4 (52.6–62.4)	0.231
BMI (kg/m^2^)	22.3 (19.9–23.8)	22 (19.4–23.4)	22.5 (20.2–23.9)	0.339
Breast cancer stage, *n* (%)				0.984
1	29 (39.7)	13 (38.2)	15 (38.5)	
2	39 (53.5)	20 (58.9)	20 (51.3)	
3	5 (6.8)	1 (2.9)	4 (10.2)	
Hypertension, *n* (%)				0.093
Yes	5 (6.8)	2 (5.9)	3 (7.7)	
No	68 (93.2)	32 (94.1)	36 (92.3)	
Diabetes mellitus, *n* (%)				0.091
Yes	6 (8.2)	5 (14.7)	1 (2.6)	
No	67 (91.8)	29 (85.3)	38 (97.4)	
Smoking, *n*	0	0	0	
Alcohol, *n*	0	0	0	
Family history of breast cancer, *n* (%)				0.725
Yes	9 (12.3)	5 (14.7)	4 (10.3)	
No	64 (87.7)	29 (85.3)	35 (89.7)	
Chemotherapy regimen, *n* (%)				0.752
E + C + P	53 (72.6)	26 (76.5)	27 (69.2)	
E + C + T	11 (15.1)	5 (14.7)	6 (15.4)	
E + C	9 (12.3)	3 (8.8)	6 (15.4)	
ST2 (baseline) (ng/ml)	11.5 (7.0–13.1)	11.8 (7.5–14.9)	11.1 (6.1–12.1)	0.262
NT-proBNP (baseline) (pmol/L)	5.7 (2.3–7.6)	4.2 (2.1–5.4)	5.6 (3.1–9.3)	0.016
hs-cTnT (baseline) (ng/ml)	0.004 (0.003–0.005)	0.004 (0.003–0.006)	0.004 (0.003–0.005)	0.719
H-FABP (baseline) (ng/ml)	1.8 (1.3–1.9)	1.5 (1.2–1.9)	1.8 (1.3–2)	0.336

BMI, body mass index; EC-P, epirubicin + cyclophosphamide + paclitaxel; EC-T, epirubicin + cyclophosphamide + docetaxel; EC, epirubicin + cyclophosphamide; NT-proBNP, N-terminal pro-B-type natriuretic peptide; sST2, soluble ST2 (growth stimulation-expressed gene 2); hs-cTnT, high-sensitivity cardiac troponin T; H-FABP, heart-type fatty acid binding protein.

Data are median (interquartile range) or *n* (%). *P*-value: normal vs. arrhythmia.

All the patients received four courses of anthracycline (epirubicin) and alkylating-based (cyclophosphamide) (EC) chemotherapy followed by 9–12 weeks of tubulin-binding agent (paclitaxel or docetaxel)-based chemotherapy. The study protocol conforms to the ethical guidelines of the 1975 Declaration of Helsinki as reflected in *a priori* approval by Fudan University Shanghai Cancer Center Ethics Committee.

### 2.2 Blood samples collection

**Figure 1:**
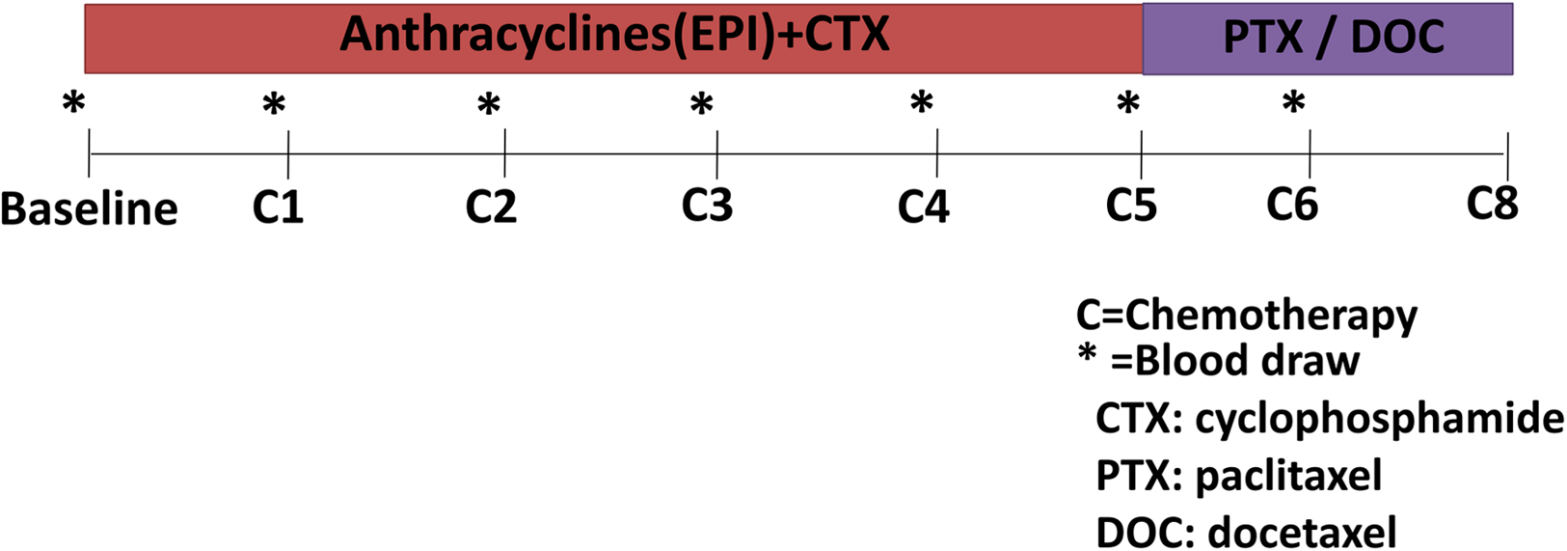
Timeline of blood draws according to cancer therapy regimen.

Clinical data were collected before treatment initiation from inpatient medical records. Blood samples were drawn at standardized time intervals according to the cancer therapy regimen (Figure 1) (20). The blood samples were collected at baseline, before every cycle of EC chemotherapy (C1–C4), and during the paclitaxel or docetaxel treatment (≈5 months, C5; ≈6 months, C6). A 10 ml blood sample was collected and the serum was prepared in 1 h and then stored at −80℃.

### 2.3 Biomarker measurements

Six biomarkers, namely, sST2, hs-cTnT, NT-proBNP, myoglobin (MYO), creatine kinase isoenzyme MB (CKMB), and heart-fatty acid binding protein (H-FABP) were assessed in all the treatment groups. Serum hs-cTnT was assayed using the fourth-generation Elecsys on the Cobas platform (Roche Diagnostics, USA). Serum NT-proBNP, CKMB, and MYO levels were measured on the Cobas platform (Roche Diagnostics, USA). The serum sST2 and H-FABP concentrations were measured using the enhanced fluorescent immunoassay performed on a Pylon 3D immuno-analyzer (ET Healthcare, Suzhou, China).

### 2.4 Electrocardiogram detection and chemotherapy-related arrhythmias definition

The electrocardiograms were conducted using a digital work platform (SID Medical, Shanghai, China). Normal heart rate (HR) was defined as 60–100 beats per minute (BPM). Bradycardia was defined as lower than 60 beats per minute, and tachycardia was defined as higher than 100 beats per minute. Cancer therapy-related arrhythmia was differentiated into bradycardia or tachycardia, with atrial fibrillation (AF) emerging as an important complication (4). The time from baseline to arrhythmia occurrence in the arrhythmia group is shown in Supplementary Figure S3, with an average time of 83.6 days. The average HR of patients with tachycardia was 107.3 BPM. The average HR of the patients with bradycardia was 54 BPM. There was just one AF in the study population. However, two patients had first-degree atrioventricular block and two patients had ST segment deviation.

### 2.5 Statistical analysis

Baseline characteristics were described using proportions for categorical variables, and medians with interquartile range were presented for continuous variables. The chi-square test was used to compare the differences among the categorical variables. The Mann–Whitney *U* test was used to compare the continuous variables. The Wilcoxon signed-rank test was used to assess the change in each biomarker concentration from baseline to 6 months.

According to previous references (5, 21) and clinical significance, variables closely related to an adverse prognosis of cancer treatment-related cardiac toxicity were included as adjusted covariates, including age, gender, BMI, systolic blood pressure, and diabetes. A logistic regression model was used to assess associations between changes in biomarker values and cardiotoxicity. Receiver operating characteristic (ROC) curves were used to evaluate the efficacy of the model. All of the tests were two-tailed, and a value of *P* < 0.05 indicated statistical significance. All analyses were performed by SPSS 20.2 and Medcalc 20.0.

## 3 Results

### 3.1 Basic clinical characteristics of the study population

The baseline characteristics and biomarker values are shown in Table 1. A total of 73 patients were enrolled as a prospective cohort. The participants were divided into two groups based on the electrocardiogram results. Thus, 39 patients were placed in the arrhythmia group (*n* = 39, median age = 44.3 years, range = 35.5–53.5 years), and the normal group comprised 34 patients (*n* = 34, median age = 48.7 years, range = 43–55 years). At baseline, the level of sST2 in the total population was 11.5 (7.0–13.1) ng/ml and the level of NT-proBNP in the total population was 5.7 (2.3–7.6) pmol/L. Compared to the normal group, the baseline median NT-proBNP levels of the arrhythmia group were higher (*P* = 0.016). The two groups of patients had no significant differences in age, weight, BMI, breast cancer stage, chemotherapy regimen, sST2, hs-cTnT, or H-FABP (all *P* > 0.05).

In the relatively normal group in this study, just 10 patients underwent echocardiography at baseline and the end of chemotherapy treatment, while only four patients had echocardiography results at the end of the treatment. The LVEF results were all greater than 50%, and there was no 10% decline after chemotherapy (as shown in Supplementary Figure S1). In the arrhythmia group, 14 patients underwent echocardiography at the baseline and the end of chemotherapy treatment, but 8 patients only had echocardiography results at the end of chemotherapy. Meanwhile, the LVEF values were greater than 50% and no 10% decline occurred after chemotherapy (as shown in Supplementary Figure S1).

### 3.2 Biomarker trends during the cancer chemotherapy process

**Figure 2:**
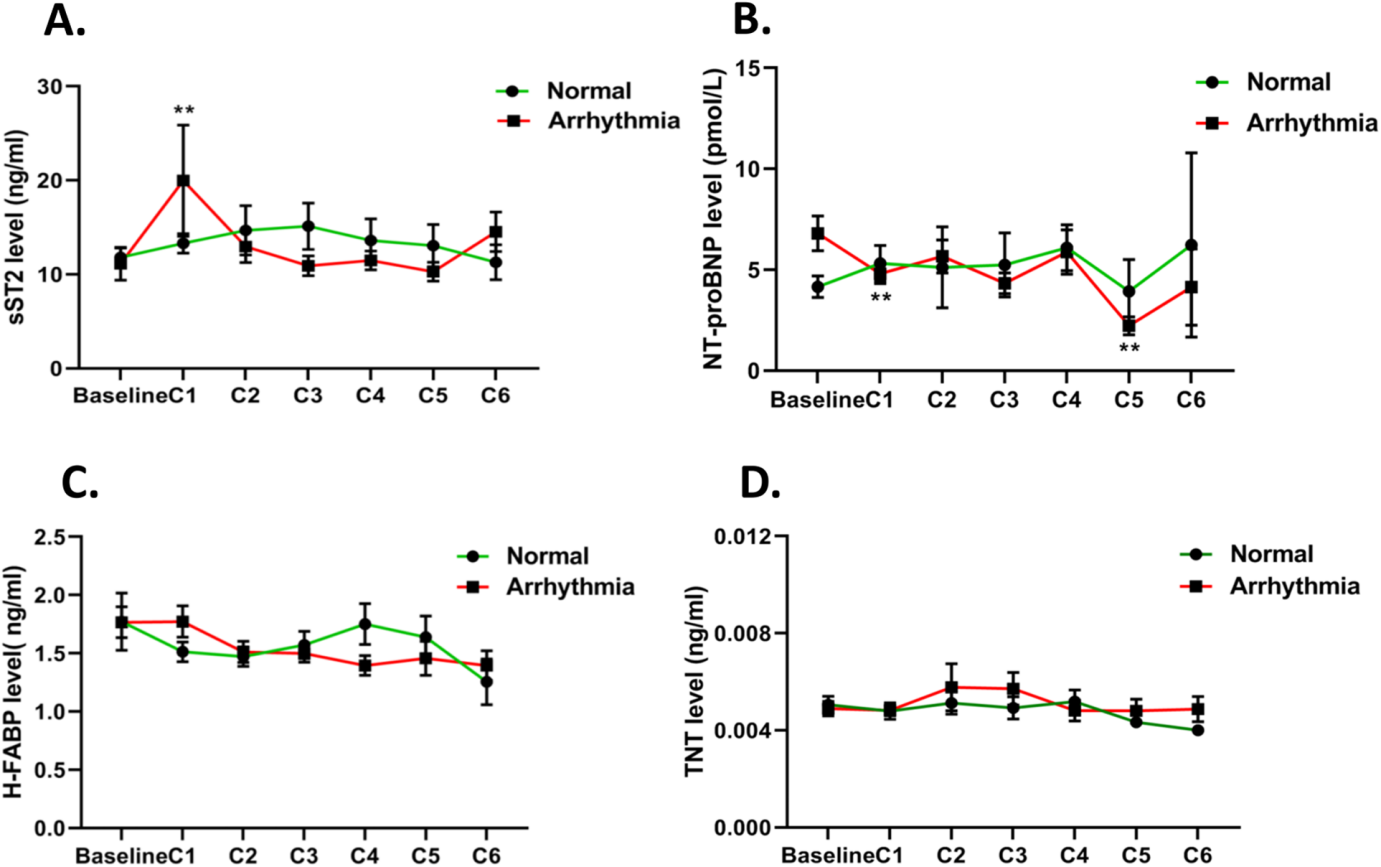
Mean estimated changes in biomarkers over time according to cancer therapy regimens. The red line represents the mean estimated changes in biomarkers in the arrhythmia group and the green line represents the changes in biomarkers in the normal group: **(A)** sST2, **(B)** NT-proBNP, **(C)** H-FABP, and **(D)** hs-cTnT. *P < 0.05; **P < 0.01.

The dynamic changes in serum biomarkers during the cancer chemotherapy process are presented in Figures 2A–D and Supplementary Figure S2. The sST2 level significantly increased in the arrhythmia group, ≈1.6-fold (*P* < 0.05) from baseline to the first EC cycle (C1, ≈21 days), and then decreased to baseline level from C2 to C6, while sST2 levels remained at baseline or were slightly increased throughout the follow-up in the normal group (Figure 2A). The NT-proBNP level demonstrated an early, significant decrease in the arrhythmia group at C1 compared to baseline (*P* < 0.01). Furthermore, the NT-proBNP levels had a more significant decrease at C5 (*P* < 0.01). However, there was no significant change in NT-proBNP levels in the normal group observed, except for a slight decrease at C5 (Figure 2B). The serum H-FABP levels decreased slightly and gently in both groups (Figure 2C), but had no significant differences. In addition, there were no significant changes in the hs-cTnT levels across the chemotherapy process (Figure 2D). Meanwhile, the traditional cardiac biomarkers MYO and CKMB had no significant changes in both groups (Supplementary Figure S2).

### 3.3 Association between the changes in biomarker levels and arrhythmia

**Figure 3:**
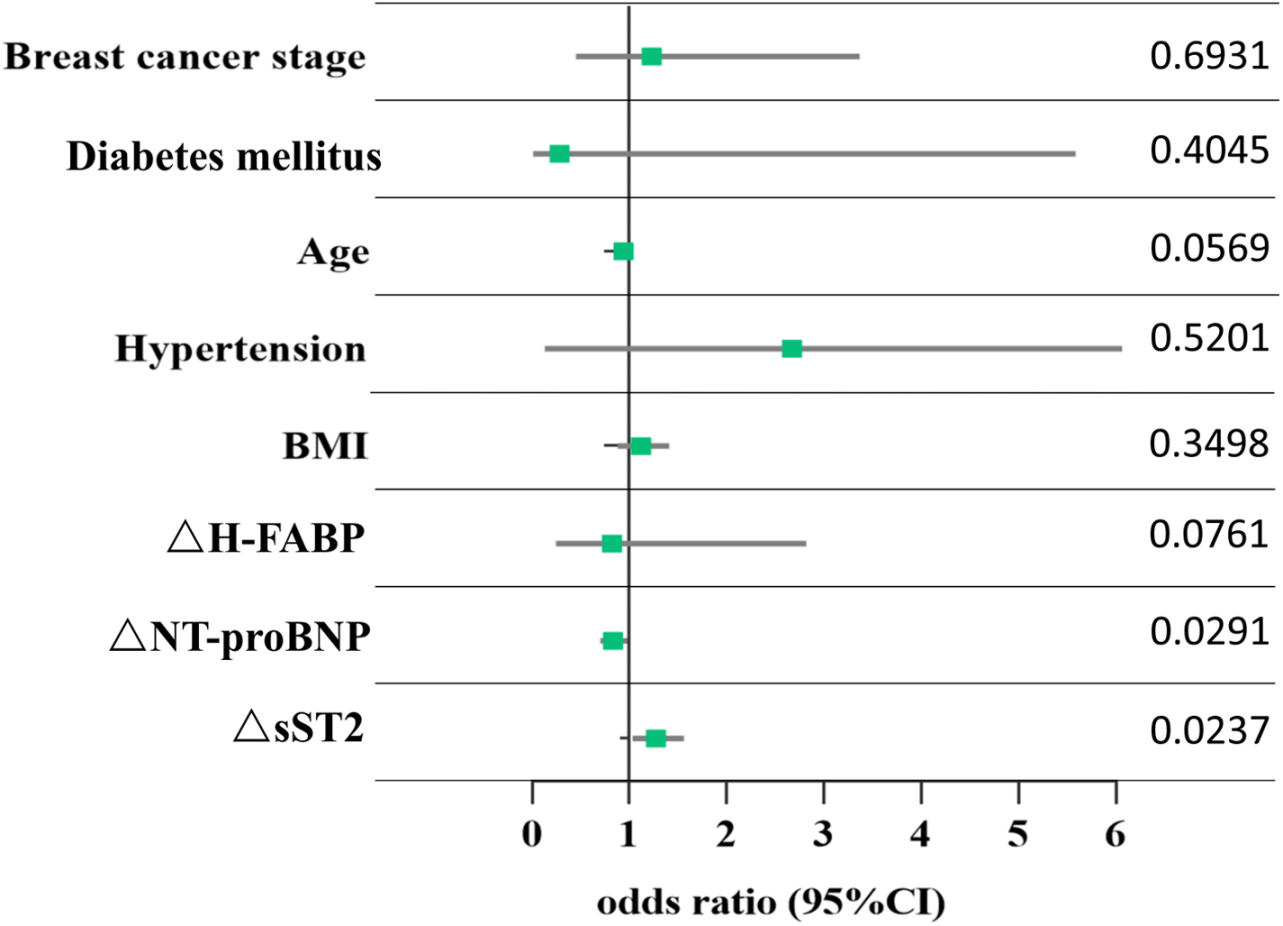
Association between changes in biomarkers and arrhythmia. Each point corresponds to the occurrence of arrhythmia. ΔsST2, ΔNT-proBNP, and ΔH-FABP represent the change in the levels of sST2, NT-proBNP, and H-FABP from baseline to chemotherapy cycle 1, respectively. The data show the *P-*value of each point.

A total of 39 participants (53.4%) were defined as having cardiotoxicity. The results of the multivariate logistic regression analysis showed that interval changes in sST2 (ΔsST2) and NT-proBNP (ΔNT-proBNP) from baseline to C1 were independent predictors of arrhythmia in patients with breast cancer (Table 2). For each increase in sST2 (a change of 1 ng/ml), there was a 27% increased risk of cardiotoxicity [odds ratio (OR): 1.27; 95% CI: 1.03–1.56; *P* = 0.024]. A significant association was observed for NT-proBNP (OR: 0.83; 95% CI: 0.70–0.98; *P* = 0.029). Compared to baseline, for each 1 unit change in NT-proBNP level, the risk of arrhythmia in patients was reduced by 17%. The odds ratios (95% CI) and *P*-values are shown in Figure 3.

**Table 2:** Associations between changes in biomarker values and arrhythmia.

	Univariate			Multivariate		
	OR (95% CI)	*β*	*P*-value	Adjusted OR (95% CI)	*β*	*P*-value
Age	0.96 (0.91–1)	−0.044	0.0729	0.94 (0.89–1)	−0.058	0.0569
BMI	1.08 (0.91–1.27)	0.075	0.381	1.12 (0.88–1.41)	0.112	0.3498
Hypertension	1.33 (0.21–8.49)	0.29	0.7607	2.67 (0.13–53.46)	0.983	0.5201
Diabetes mellitus	0.15 (0.02–1.38)	−1.88	0.0941	0.28 (0.01–5.59)	−1.273	0.4045
Breast cancer stage	1.2 (0.56–2.57)	0.18	0.6382	1.23 (0.45–3.37)	0.203	0.6931
ΔsST2	1.16 (1.01–1.34)	−0.29	0.0371	1.27 (1.03–1.56)	0.237	0.0237
ΔNT-proBNP	0.85 (0.74–0.97)	−0.16	0.02	0.83 (0.70–0.98)	−0.192	0.0291
ΔH-FABP	1.31 (0.75–2.34)	0.28	0.3362	0.82 (0.24–2.82)	−0.195	0.7561
Δhs-cTnT	—	381.36	0.0647	—	400.41	0.0880

### 3.4 Predictive value of ΔsST2 and ΔNT-proBNP for arrhythmia

**Figure 4:**
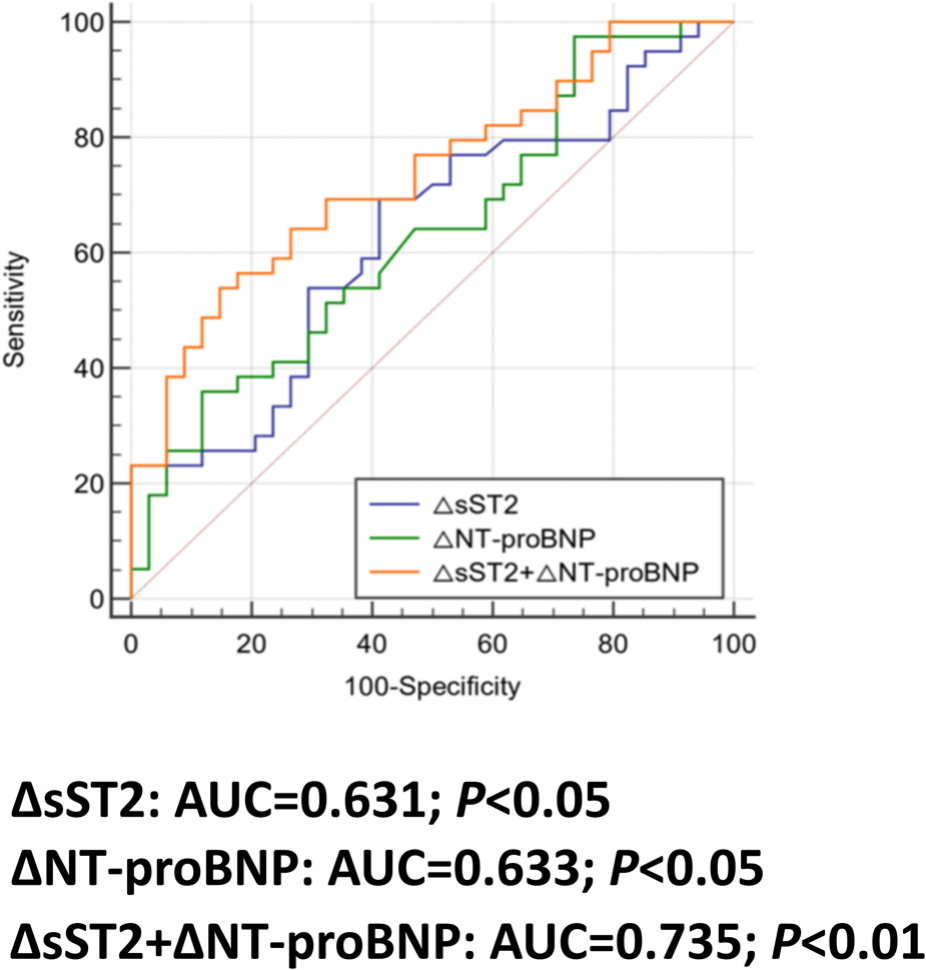
The area under the curve (AUC) for predicting arrhythmia. ROC curve analysis of ΔsST2/ΔNT-proBNP from baseline to C1 predicts the occurrence of arrhythmia. The combination of ΔsST2 and ΔNT-proBNP is also analyzed.

The ROC curves (Figure 4) showed that ΔsST2 and ΔNT-proBNP had predictive value for arrhythmia. The areas under the curves (AUCs) were 0.631 and 0.633, respectively (*P* < 0.05). Moreover, the combination of ΔsST2 + ΔNT-proBNP significantly increased the predictive value for arrhythmia, with an AUC of 0.735 (*P* < 0.01). In total, 41/73 (56.2%) were above the cutoff value for ΔsST2, and elevations above the threshold had 69.23% sensitivity and 58.82% specificity for the prediction of arrhythmia, with accompanying positive predictive value (PPV) and negative predictive value (NPV) of 65.9% and 62.5%, respectively (Table 3). Furthermore, 18/73 (24.7%) were below the cutoff value for ΔNT-proBNP, and a change of ΔNT-proBNP <−2.16 had 35.90% sensitivity and 88.2% specificity, with accompanying PPV and NPV of 77.8% and 54.5% (Table 3). When ΔsST2 + ΔNT-proBNP were combined, there was significantly higher specificity (85.29%) and PPV (80.8%) (*P* = 0.0001) (Table 3).

**Table 3:** ROC curves analyses of the predictive performance of sST2 and NT-proBNP for arrhythmia.

Cutoff value	Sensitivity (%)	Specificity (%)	AUC	PPV (%)	NPV (%)	*P-*value
ΔsST2						
1.53	69.23	58.82	0.631	65.9	62.5	0.0472
ΔNT-proBNP						
−2.16	35.90	88.24	0.633	77.8	54.5	0.0413
ΔsST2 + ΔNT-proBNP	53.85	85.29	0.735	80.8	61.7	0.0001

### 3.5 Serum sST2 and NT-proBNP levels predict early arrhythmia

**Figure 5:**
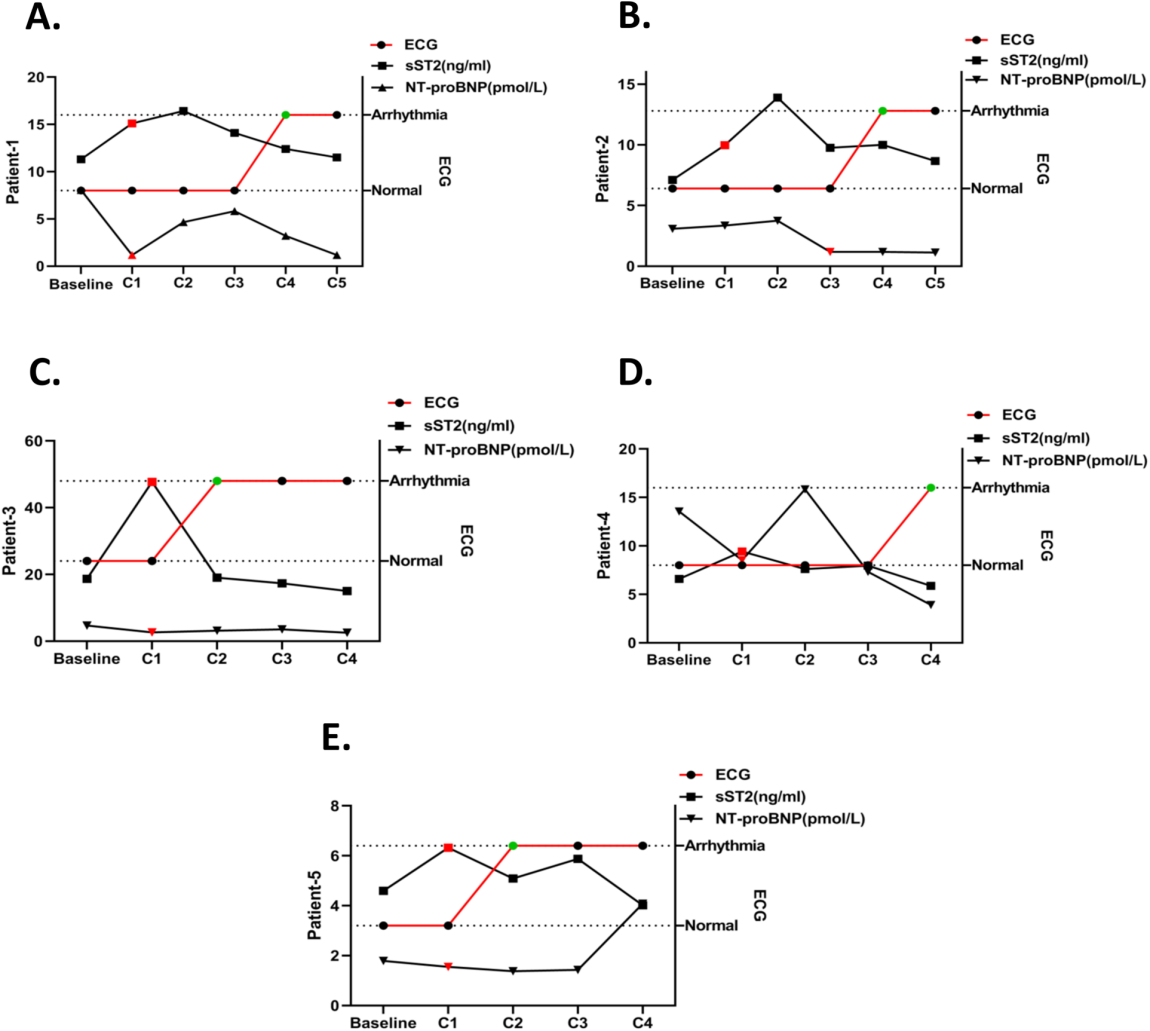
Timeline of sST2/NT-proBNP level changes and ECG abnormalities in arrhythmia patients. The timelines of sST2 and NT-proBNP changes in one arrhythmia patient are shown and the red plot represents the abnormal change point. ECG detection time point is shown and the green plot represents the abnormal change point. **(A)** Patient 1, **(B)** patient 2, **(C)** patient 3, **(D)** patient 4, and **(E)** patient 5.

To explore whether the changes in the serum biomarkers sST2 and NT-proBNP occurred earlier than ECG abnormalities, timelines of the patients were created, as shown in Figure 5. In patient 1, their sST2 level increased and NT-proBNP level decreased significantly in the C1 period, while an abnormal ECG was observed until C4 (Figure 5A). The sST2 level of patient 2 increased in the C1 period and NT-proBNP decreased at C3, while ECG abnormality occurred at C4 (Figure 5B). The sST2 and NT-proBNP levels of patient 3 significantly changed at C1, but ECG changed at C2 (Figure 5C). In patient 4, significant changes in sST2 and NT-proBNP appeared at C1, while ECG abnormalities appeared until C4 (Figure 5D). Serum sST2 level increased and NT-proBNP level decreased at C1 for patient 5, while the ECG changed until C2 (Figure 5E).

## 4 Discussion

To our knowledge, this is the first time each time point of the treatment cycle of an anthracycline-based regimen was monitored from baseline to C1–C6, and the blood samples were collected as early as 1 month after treatment. The levels of biomarkers sST2, NT-proBNP, cTnT, H-FABP, CKMB, and MYO were detected at each point of the therapy cycle. In contrast, previous studies started monitoring blood biomarkers at least 2 or 3 months after treatment (22, 23). In addition, an exploration of the association between multiple biomarkers and cardiotoxicity during an EC-P/T chemotherapy regimen is rare.

This study showed that the sST2 levels significantly increased early in the C1 period compared to baseline, while NT-proBNP levels decreased at C1 and C5 compared to baseline in the arrhythmia group. Logistic regression analysis showed that interval changes in sST2 and NT-proBNP levels were associated with a greater risk of arrhythmia. Meanwhile, the ROC curves showed that ΔsST2, ΔNT-proBNP, and ΔsST2 + ΔNT-proBNP had good predictive values for arrhythmia.

In this study, the electrocardiograms of the study participants were constantly monitored and arrhythmia was identified as cardiac toxicity. Early changes in electrocardiograms caused by anthracycline drugs are generally reversible, but may also manifest as myocardial injury and eventually progress to worse cardiac damage such as reduced heart function and heart failure. Early identification and monitoring are essential (4). Other studies confirmed that cardiac cell injury can occur during the first application of anthracycline drugs with subclinical symptoms that may develop into LVEF decline or heart failure (24, 25). The cardiac toxicity of cancer treatment resulting in arrhythmias is not well recognized (26). Anthracycline drugs are closely related to various arrhythmias, including malignant arrhythmias. Cancer patients who receive anthracycline may have significantly prolonged ventricular repolarization indicators (such as the QT interval, i.e., the measure of the time between the start of the Q wave and the end of the T wave in the heart's electrical cycle, and QTc, the corrected QT interval) which may be an early sign of malignant arrhythmias (27–29). Moreover, in real clinical settings, quantitative echocardiography is not performed frequently and in a timely manner due to its complexity and cost, while electrocardiograms can be used for more frequent and timely monitoring.

sST2, a biomarker for predicting heart failure and myocardial infarction, also has potential value for monitoring cancer treatment-related cardiac dysfunction (30). The individual differences in anthracycline metabolism-related genes lead to different susceptibilities to anthracycline drugs (31). In this study, sST2 increased significantly early in the first EC chemotherapy cycle and then declined to a steady level, indicating that sST2 may predict early myocardial injury in anthracycline chemotherapy prior to arrhythmias being diagnosed on electrocardiograms. Furthermore, the initial increase and then the decrease in sST2 levels were also observed in previous studies (17, 32). This study also found that the magnitude of change in sST2 levels was related to the development of cancer-related cardiotoxicity and was an independent predictor of arrhythmia after chemotherapy. Meanwhile, compared to other cardiac biomarkers, sST2 is minimally influenced by age, has the lowest intra-individual variability, and shows the smallest relative variation (33), making it suitable for monitoring treatment-related adverse reactions. The mechanism of arrhythmias caused by anthracycline drugs may be related to the activation of the Toll-like receptor (TLR)-mediated pro-inflammatory pathway (34). As a member of the Toll-like receptor family, sST2 possesses TLR structural domains, which may be the reason for the elevated serum sST2 levels after the initial chemotherapy in this study.

Moreover, this study found that NT-proBNP levels decreased significantly after initial treatment with anthracycline drugs and further declined during the C5 regimen. The early decline might be related to the patients’ higher baseline levels. Some previous studies revealed NT-proBNP levels increased in cancer treatment-induced cardiotoxicity groups (15, 23), while the time points for detecting NT-proBNP were often as early as 6 months or even later after treatment. Moreover, the transient decrease in NT-proBNP levels may be a stress reaction of myocardial cells to the initial exposure to EC chemotherapy drugs to alleviate ventricular wall pressure (35). The early decrease in NT-proBNP levels may be a self-protection mechanism of the heart to relieve stress and prevent heart failure. Furthermore, elevated NT-proBNP levels may be revealed during longer follow-up periods (36). Finally, the combined monitoring of NT-proBNP and sST2 provides incremental value for predicting the clinical risk factors for anthracycline-induced arrhythmias.

cTnTs are most widely studied as biomarkers in cardio-oncology. An elevation of cTnI levels was reported with high-dose chemotherapy and predicted the risk of cardiac toxicity (37, 38). Furthermore, previous studies showed that patients treated with anthracycline-based regimens had elevated cTnT levels (22, 23). In this study, hs-cTnT levels did not significantly change during EC-P/T treatment. The reason might be that in previous studies anthracycline regimens were primarily based on doxorubicin, which has more severe cardiac toxicity than epirubicin (39). Meanwhile, in our study population, the cardio-protective drug dexrazoxane was used throughout the EC treatment process. Thus, myocardial cells could be protected from typical apoptosis or death.

Our study confirms that sST2 and NT-proBNP are associated with arrhythmia in patients with breast cancer undergoing anthracycline-containing chemotherapy. It provided incremental data to support sST2 and NT-proBNP as potential biomarkers for arrhythmia risk prediction. In addition, the sST2 increase and NT-proBNP decrease in the initial treatment phase were associated with arrhythmia. More importantly, we found that the time points at which the sST2 and NT-proBNP levels changed were earlier than the arrhythmia detection time points by electrocardiogram. Multiple biomarkers may enhance the sensitivity of arrhythmia risk prediction in patients treated with an anthracycline regimen.

## 5 Study limitations

The study also had some limitations. Our cohort comprised 73 patients, of which 39 patients with arrhythmia were defined as the arrhythmia group. Even though we monitored each point of the chemotherapy process, the overall number of participants was still small. The small cohort number limits our ability to differentiate the biomarker associations with acute or delayed recovery. In a future study, we will expand the cohort and establish a training test to further validate our results.

## 6 Conclusions

Our study confirms that sST2 and NT-proBNP level changes in the early cycle of an anthracycline-containing regimen were associated with arrhythmia. The time points at which the sST2 and NT-proBNP levels changed were earlier than arrhythmia detection time points by electrocardiogram. The multi-biomarker approach may increase the sensitivity of arrhythmia prediction in breast cancer patients treated with anthracyclines.

## Data Availability

The raw data supporting the conclusions of this article will be made available by the authors, without undue reservation.
