# Supplementary figures and images for: Changes in sST2 and NT-proBNP levels predict early cardiac arrhythmia in breast cancer patients treated with anthracycline-containing chemotherapies
Source: Front Cardiovasc Med. 2024 Dec 12;11:1477679. doi: 10.3389/fcvm.2024.1477679 (PMC11669546; doi:10.3389/fcvm.2024.1477679)

Supplementary Figure 1

A.

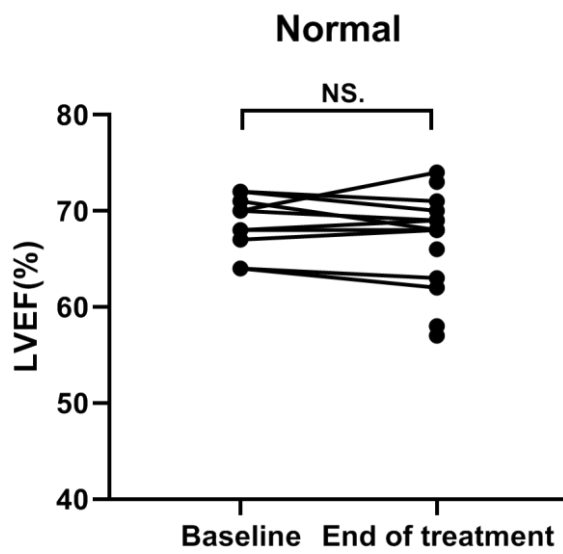

B.

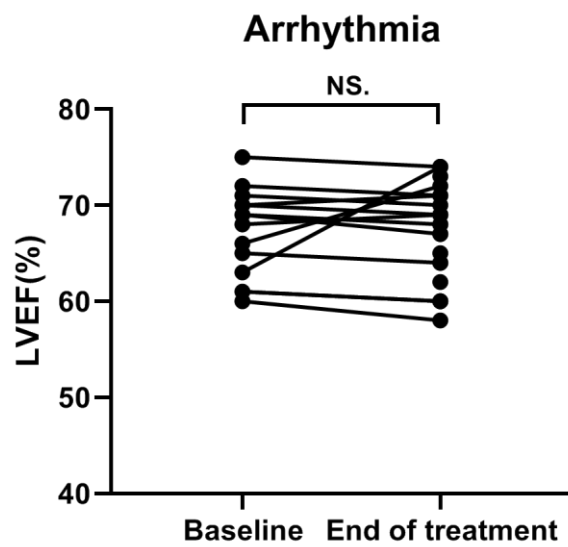

Supplementary Figure 2

A.

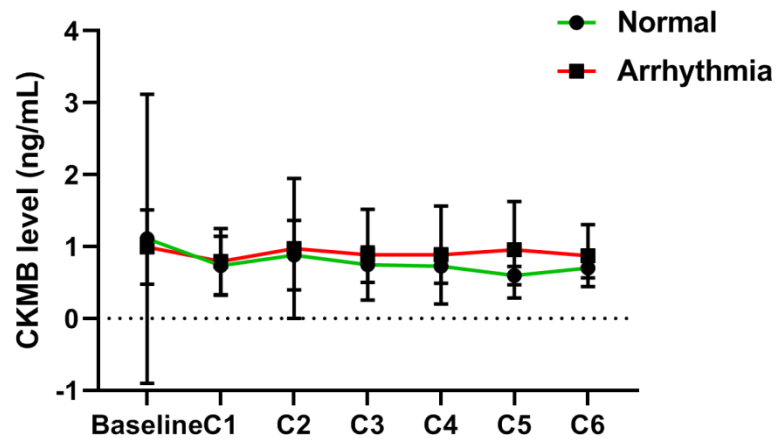

B.

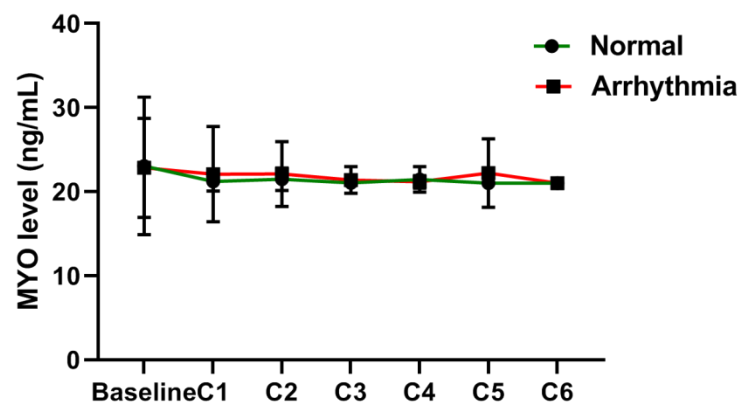

Supplementary Figure 3

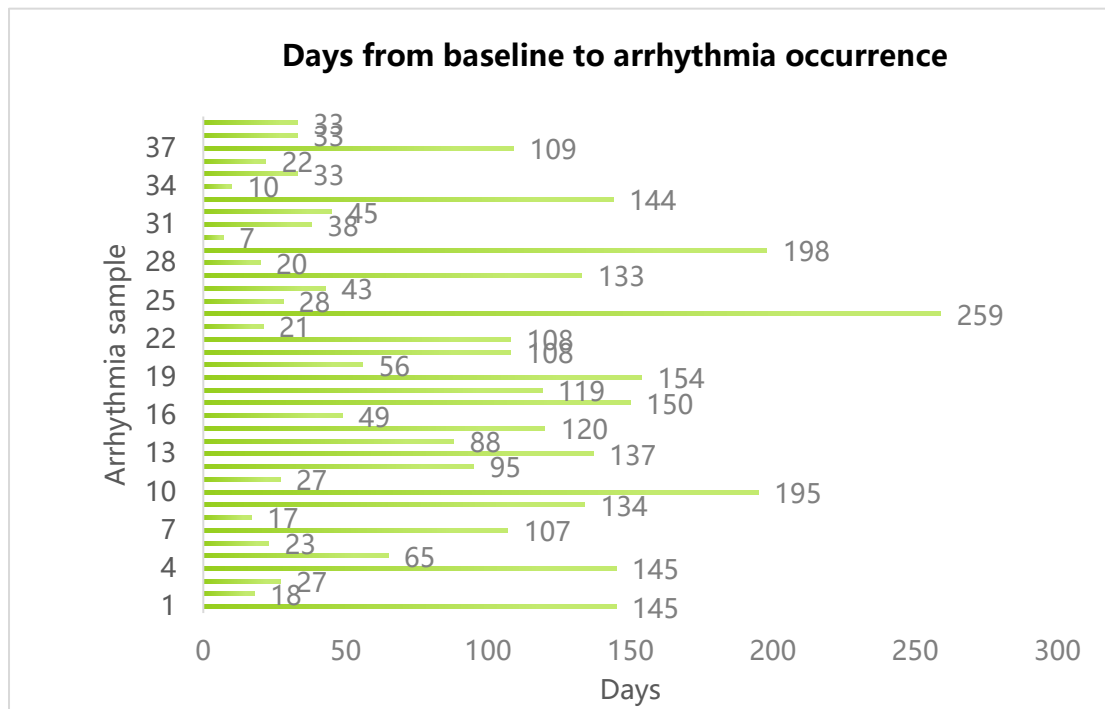

Supplement: Supplementary file 1 [file Supplementaryfigures.pdf]
